# Polyphenols and Sunburn

**DOI:** 10.3390/ijms17091521

**Published:** 2016-09-09

**Authors:** Suzana Saric, Raja K. Sivamani

**Affiliations:** 1 School of Medicine, University of California, Davis, Sacramento, CA 95817, USA; ssaric@ucdavis.edu; 2 Department of Dermatology, University of California, Davis, Sacramento, CA 95816, USA

**Keywords:** polyphenols, flavonoids, antioxidant, sunburns, UVA, UVB, skin damage

## Abstract

Polyphenols are antioxidant molecules found in many foods such as green tea, chocolate, grape seeds, and wine. Polyphenols have antioxidant, anti-inflammatory, and antineoplastic properties. Growing evidence suggests that polyphenols may be used for the prevention of sunburns as polyphenols decrease the damaging effects of ultraviolet A (UVA) and ultraviolet B (UVB) radiation on the skin. This review was conducted to examine the evidence for use of topically and orally ingested polyphenols in prevention of sunburns. The PubMed database was searched for studies that examined polyphenols and its effects on sunburns. Of the 27 studies found, 15 met the inclusion criteria. Seven studies were conducted on human subjects and eight on animals (mice and rats). Eleven studies evaluated the effects of topical polyphenols, two studies examined ingested polyphenols, and two studies examined both topical and ingested polyphenols. Polyphenol sources included the following plant origins: green tea, white tea, cocoa, Romanian propolis (RP), *Calluna vulgaris* (Cv), grape seeds, honeybush, and *Lepidium meyenii* (maca). Eight studies examined green tea. Overall, based on the studies, there is evidence that polyphenols in both oral and topical form may provide protection from UV damage and sunburn, and thus are beneficial to skin health. However, current studies are limited and further research is necessary to evaluate the efficacy, mechanism of action, and potential side effects of various forms and concentrations of polyphenols.

## 1. Introduction

Ultraviolet A (UVA) and ultraviolet B (UVB) rays are damaging to the skin. Aside from aging with the passage of time, skin may also age prematurely as a result of exposure to UVA and/or UVB light [1]. Direct exposure to UVB radiation leads to DNA disruption [2]. As a result of overexposure to UVA or UVB light, skin begins to lose rigidity and elasticity, appearing wrinkly and rough to the touch [3]. Recent research has shown elevated levels of mitochondrial DNA mutations in prematurely aged skin following habitual exposure to UV light [1]. The most effective protection is avoidance of exposure to UVB radiation (the sun), but this approach is not practical in everyday life. However, polyphenols may provide a more practical solution to protecting the skin from UVB radiation.

Polyphenols are antioxidant molecules that, like antioxidant vitamins and enzymes, help prevent the oxidative stress caused by excessive reactive oxygen species (ROS) [4]. The antioxidant properties of polyphenols are primarily due to the presence of hydroxyl groups [2]. Typically, polyphenols are ingested and then deglycosylated and absorbed across the intestinal epithelium [5]. There is increasing evidence for the bioavailability of polyphenols, once ingested, in systemic circulation [6,7,8,9]. In a study by Clarke et al. [10], systemic green tea was provided in the form of capsules with a polyphenol content of 180 mg catechins in each capsule. The total amount ingested was equal to five cups of green tea daily, with a total polyphenol content of 1080 mg green tea catechins daily. This was the first study that identified green tea catechin conjugates and their metabolites in plasma, blister fluid, and skin biopsy samples. Besides their antioxidant properties, polyphenols may also act as enzyme inhibitors or inducers, impacting anti-inflammatory pathways [1]. Polyphenols may be found in everyday, common foods such as green tea, chocolate, and red wine.

Current research suggests that polyphenols may be an effective source of skin protection from the effects of UV radiation (UVA and UVB) [1]. Application and consumption of different types of polyphenols has been shown to lead to lower UVB-caused skin sunburn. This review will describe the recent research on the effects of the use of sources of polyphenols in protecting the skin from UVB radiation (sunburn).

## 2. Results

Of the 27 studies found, 15 met the inclusion criteria. Seven studies were conducted on humans and eight on animals. Eleven studies examined topical polyphenols, two studies examined systemic polyphenols, and two studies examined both topical and systemic polyphenols. Polyphenols studied included the following plant origins: green tea, white tea, cocoa, Romanian propolis (RP), *Calluna vulgaris* (Cv), grape seeds, honeybush, and *Lepidium meyenii* (maca). Eight studies examined green tea.

### 2.1. Green Tea

Studies have shown a correlation between green tea consumption and decreased risk of cancer and cardiovascular disease [11], as well as skin protection from ultraviolet radiation (UVR) [12,13,14]. Green tea contains flavonoids called catechins, which include catechin (C), epicatechin (EC), epigallocatechin (EGC), and epigallocatechin gallate (EGCG) [10]. After consumption of green tea, catechins undergo phase II metabolism and have been shown to be present in conjugated and unconjugated forms in plasma [14,15]; they have also been identified in many tissues [16].

#### 2.1.1. Human Studies

In a 34-day study by Mnich et al. [17], 18 people aged 21 to 71 applied green tea topically on one side of their buttocks and a placebo topical on the other; the areas were then exposed to UVB on days 5 and 33 and erythema quantified on days 6 and 34. Skin biopsies followed. On day 34, the green tea topical pre-treated area had a 38.9% decrease in the amount of sunburn cells, which was shown to be statistically significant. These results indicate that a green tea extract topical (called OM24) is suitable for protection from UVR and sunburn.

In a study by Elmets et al. [18], subjects between 18 and 50 years old applied various green tea extract concentrations on their skin, ranging from 0.25% to 10%. This study showed that green tea polyphenol (GTP) applied before UV exposure decreased sunburn cells by 66%. The 2.5% GTP concentration provided excellent protection but beneficial effects were seen even with the lower dose of 0.5% GTP. In the second part of the study, skin was treated with equal concentrations of 5% GTP and its constituents EGCG, EC, and EGC. The results showed that 5% GTP was the most effective in protecting from erythema, and sunburn cells were reduced by 68% (*p* < 0.01). DNA damage was also reduced by 55% (*p* < 0.01). One limitation of this study is the small participant pool as only five to six volunteers participated in each part of the study. Also, this study mostly focuses on UVA light.

A double-blind, randomized, placebo-controlled trial using systemic green tea was conducted by Farrar et al. [19] in the United Kingdom in 2015. The study had 50 volunteers aged 18–65 who were randomly assigned to one of two groups: group 1 (G1) received 1080 mg/day of green tea catechins (GTC) in the form of capsules plus 100 mg/day vitamin C (to help with GTC stabilization in the gut); group 2 (G2) received placebo capsules that looked identical to G1. Before systemic GTC treatment and 12 weeks post-treatment, buttock skin was exposed to UVR and 24 h post-exposure the skin was examined visually for erythema. The outcome measure was minimal erythema dose (MED) (the lowest UV dose that produced visually detectable erythema, also known as the sunburn threshold) at baseline and 12 weeks post-treatment. The results showed no difference in MED between GTC group and placebo group (*p* = 0.47). Within the GTC group, there was no difference in MED pre- and 12 weeks post-treatment (*p* = 0.17). Additionally, the placebo group also showed no change in MED at 12 weeks compared to the baseline. This study failed to demonstrate that systemic GTC may protect against UVR-induced sunburn. Some of the reasons for this finding may include inadequate GTC dose and variable amounts of EGCG and other catechins compared to other green tea (GT) studies. Future studies might compare various dosages of systemic green tea.

Camouse et al., 2009 [20] conducted a randomized, double-blind, controlled trial to assess whether green and white tea extracts could prevent UVR-induced Langerhans cell and DNA damage, which could lead to suppression of the immune system and development of skin cancer. The trial was done in the United States and included 10 subjects (age not reported) for a duration of 72 h. There were five treatment groups: G1: No UV, no treatment; G2: UV only, no treatment; G3: vehicle + UV; G4: white tea (WT) + UV; and G5: GT + UV. Topicals were applied to the buttock in the amount of 2.5 mg/cm^2^ and allowed to dry for 15 min; UVR was then applied at twice the MED and the respective topical was applied again. Biopsies from the five sites were collected 72 h after UVR. The major outcome measures were Langerhans cells in the epidermis (detected via anti-CD1a immunostaining) and DNA damage (detected by anti-8-hydroxy-2′-deoxyguanosine (OHdG) staining). The results showed that, compared to G1, G2 and G3 each had a 57% reduction in Langerhans cells. This suggests that the topical vehicle provides no protection against LC damage and reduction. Compared to G3, G4 (*p* = 0.002) and G5 (*p* = 0.003) had a significantly higher percentage of CD1a staining, suggesting a higher amount of Langerhans cells per unit area of epidermis. Compared to G1, which did not receive UVR or any treatment, G4 and G5 had a depletion of LC (22% and 35%, respectively). Additionally, there was no difference between G4 and G5 in protection against LC depletion (*p* = 0.09) suggesting the similar efficacy of WT and GT extracts. The result examining DNA damage showed that, compared to G1, G2 had a 40% increase and G3 a 69% increase in DNA damage. The DNA damage in G4 and G5 was not significantly different than in G1 (*p* = 0.95 and *p* = 0.12). However, compared to G3, which was treated with vehicle and UVR, G4 and G5 had significantly lower 8-hydroxy-2′-deoxyguanosine (8-OHdG) (*p* = 0.002 and *p* = 0.001), representing less DNA damage when skin is treated with WT or GT topicals. There was no difference in skin protection from DNA damage between WT and GT. Overall, this study demonstrated that topical WT and GT may protect the skin from UV damage by preventing LC depletion and DNA damage that may be induced by UVR.

Li et al. [21] conducted a controlled trial in 2009 with 20 women in China to examine whether 2%–5% green tea extract (GTE) topicals protect the skin from UVR-induced photoaging and photoimmunosuppresion. Seven sites on dorsal skin were treated as follows: site 1—no UVR; site 2—UVR only; site 3—vehicle cream + UVR; site 4—2% GTE + UVR; site 5—3% GTE + UVR; site 6—4% GTE + UVR and site 7—5% GTE + UVR. Sites 3 to 7 involved application of topicals 30 min before UVR and 6, 24, and 48 h after last UVR. Biopsies from all sites were obtained 72 after last UVR. Erythema was assessed via photographs of different sites. Other outcome measures included: thickness of stratum corneum (TSC) and epidermis (TE), measured by microscopy; level of cytokeratins (CK): CK5/6 and CK 16 as well as metalloproteinases (MMP): MMP-2 and MMP-9 (scored 0–3) and density of Langerhans cells (LC), determined by immunohistochemistry. On day 1, site 5 had the least erythema while sites 2, 3, and 7 had the most and it worsened with subsequent UVR exposures. Additionally, on day 7 sites 2, 3, and 7 had post-inflammatory hyperpigmentation (PIH), sites 4 and 6 moderate PIH, and site 5 mild PIH. Sites 2 and 3 had a significant increase in TSC and TE after UVR. Compared to site 2, only sites 4 and 5′s topical treatments prevented TE rise (*p* < 0.05). Compared to site 1, sites 2, 3, 6, and 7 had increased TSC (*p* < 0.05), while sites 4 and 5 were protected. In regards to CK5/6 and CK16—sites 2 and 3 had elevation of both after UVR and sites 4, 5, 6, and 7 had significant protection, with site 5 having the most protection. Additionally, MMP-2 and MMP-9 were slightly expressed at site 1, with high expression at sites 2, 3, and 7, and significant reduction in both at sites 4, 5, and 6 (*p* values not reported). Langerhans cells were decreased by the following percentages: site 2—75%, site 3—64%, site 4—58%, site 5—46%, site 6—65%, and site 7—71%. The difference in LC reduction between site 1 and the others was significant (*p* < 0.05), but not between site 1 and 5. Overall, this study was able to show that GTE can protect the skin from damaging UVR and the 3% GTE was the most effective. MMP-2 and MMP-9 are implicated in photoaging and development of cancer and this study showed that 2% and 3% GTE inhibited expression of MMP-2 and MMP-9 post-UVR. LCs are dendritic cells found in the epidermis and the study showed that 3% GTE was protective against LC depletion post-UVR exposure. The protective effects of GTE are not dose-dependent as 3% GTE was shown to provide more skin protection than 4% or 5% GTE.

#### 2.1.2. Animal Studies 

**Figure 1:**
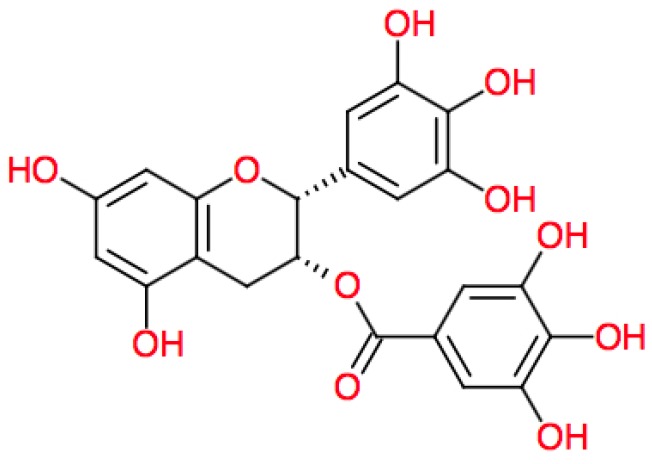
Chemical structure of (−)-epigallocatechin-3-gallate (EGCG).

Meeran et al. [22] studied the use of (−)-epigallocatechin-3-gallate (EGCG) (Figure 1), a major polyphenol in green tea, in 60 wild-type and IL-12 knockout (IL-12 KO) mice with 20 mice in each of the three treatment groups: (1) no EGCG treatment and no UVB exposure; (2) no EGCG treatment but exposed to UVB; and (3) treatment with EGCG before UVB.

Meeran et al. [22] found that topical application of EGCG prevented skin tumor incidence and multiplicity in wild-type mice, but did not prevent photocarcinogenesis in IL-12 KO mice. The number of sunburn cells decreased faster in wild-type mice treated with EGCG than in mice that received no treatment. The amount of DNA damage and number of sunburn cells did not differ significantly between the IL-12 KO mice group treated with EGCG and the untreated controls. This study showed that EGCG can prevent UVB-induced tumor development and reduce DNA damage and the amount of sunburnt cells via an IL-12-dependent mechanism.

Conney et al. [23] studied the topical and oral use of green tea in 60 mice and development of skin tumors. Topical application of 3.6 mg green tea twice per week decreased skin tumors by 94%. In the systemic part of the experiment, mice were drinking water with gradually increasing green tea concentrations; they reached 100% green tea by day 6 and continued to drink 100% green tea for a total of 25 weeks. There were six groups of 10 mice each that were placed under UV light (UVA 20%–25%, UVB 75%–80%). The number of skin tumors was significantly decreased at week 15 (*p* < 0.05) and week 25 (*p* < 0.05). In experiments 1 and 2, the intensity and severity of sunburn lesions were significantly lower in groups pretreated with green tea before UVB exposure compared to the control.

Sevin et al. [24] studied the topical application of 2% EGCG in 24 rats, applied 30 min before or after UVA exposure. The group treated with 2% EGCG 30 min before UVA exposure had a significant decrease in the number of sunburn cells at 24 h compared to the group that was not pretreated with 2% EGCG (*p* < 0.05). Limitations of this study include the small sample size and the fact that this study mainly focused on UVA radiation rather than UVB.

### 2.2. Chocolate

#### Human Studies

Chocolate is a natural source of polyphenols under the subgroup of flavonols and its polyphenol properties as antioxidant and anti-inflammatory could potentially help in prevention of sunburns and other sequela of UV damage [25]. A two-group, parallel, double-blind, randomized controlled trial conducted by Mogollon et al. [25] involved 74 women, aged 28 to 51, in Canada. The trial compared the use of high-flavonol chocolate (HFC) to low-flavonol chocolate (LFC) for 12 weeks and measured skin sensitivity to UVB (expressed as minimal erythema dose, MED). The total follow-up period was 15 weeks. After 12 weeks, both the HFC and LFC group had a similar increase in MED. The study did not show a statistically significant protective effect of HFC compared to LFC on skin sensitivity to UVB. One limitation of this study is that the LFC control group received chocolate [25], and there is no control group without any chocolate intake. Future studies are needed to address the potential of chocolate polyphenols in the prevention of sunburns and should utilize a control group with no chocolate ingestion.

### 2.3. Red Wine and Grape Seeds

#### 2.3.1. Human Studies

Red wine is known to have a high polyphenol content but its potential to protect skin from UVB radiation has not been fully investigated in humans [26]. Moehrle et al. [26] conducted a controlled study with 15 healthy male physicians, aged 28 to 51, in Germany during the spring of 2002. The study investigated the topical and systemic use of three wines with different polyphenol contents. Wine A had the lowest polyphenol content while wine C had the highest polyphenol content. Ethanol (12%) served as the control. In the topical experiment, 5 mL of each wine and control alcohol was applied to the back of each individual for 20 min, after which the skin was exposed to UVB and MED was measured as a representation of erythema after 24 h. Eight volunteers completed the topical part of the experiment and there was no significant difference between MED at baseline and MED local. At least seven days were allowed between experiments. The systemic dose of wine was 6 mL wine/kg body weight over 40 min. The results showed that Wine C oral consumption resulted in a statistically significant decrease in MED systemic compared to MED at baseline (*p* = 0.031). Limitations of this study include the small sample size and lack of history about wine use prior to this experiment.

#### 2.3.2. Animal Studies

Grapes (Vitis Vinifera) are widely available across the world and their seeds are rich in polyphenols [27], which allows them to have anti-inflammatory and antioxidant effects [28]. Filip et al. [29] examined the Vitis Vinifera Burgund Mare (BM) variety in a study that included 80 mice separated into eight groups. The polyphenol content of BM was 2.5 mg polyphenols/cm^2^ and 4 mg polyphenols/cm^2^. The groups were randomized as follows: (1) control; (2) vehicle; (3) UVB exposure; (4) vehicle + UVB; (5) BM 2.5 mg polyphenols/cm^2^ + UVB; (6) BM 4 mg polyphenols/cm^2^ + UVB; (7) UVB + BM 2.5 mg polyphenols/cm^2^; and (8) UVB + BM 4 mg polyphenols/cm^2^. In skin exposed to UVB radiation there was a significant increase in the number of sunburnt cells (*p* < 0.01). Pre-treatment with either dose of BM resulted in a decrease in the number of sunburnt cells and DNA lesions.

### 2.4. Romanian Propolis (RP)

#### Animal Studies

Propolis is collected from plant resins by bees and is known as one of the richest sources of polyphenols [30]. There is high variability in propolis composition dependent on geographic area, vegetation, and methods used to determine its chemical composition [31] but RP specifically has been shown to have high antioxidant activity and significant biological effects [32]. Bolfa et al. [33] conducted a controlled study in 30 female Swiss mice to investigate the topical application of romanian propolis (RP) and its photoprotective effects against UVB. RP with two different polyphenol concentrations was used: RP1 = 3 mg and RP2 = 1.5 mg polyphenols/cm^2^. RP was applied topically three times over 24 h without UVB radiation, before UVB or after UVB. Pre-treatment with both concentrations of RP minimized the amount of sunburn cells in the skin of mice (*p* < 0.001). This study suggests that RP protects skin from UVB damage. One limitation of the study is that it did not investigate the RP use in long-term UVB exposure but RP effects are promising for topical applications.

### 2.5. Calluna vulgaris (Cv) Extract

#### Animal Studies

*Calluna vulgaris* (Cv) is used in folk medicine due to its anti-inflammatory properties, and it is known to contain polyphenols including hyperoside, quercitrin, quercetin, and kaempferol [34]. Olteanu et al. [34] conducted a study with 50 mice to investigate the effects of Cv extract on the skin of mice after exposure to UVB. The mice were divided randomly into 5 groups: (1) control (no treatment); (2) vehicle; (3) UVB exposure; (4) Cv + UVB exposure; and (5) Cv + vehicle + UVB. The results indicated an increased number of sunburnt cells (*p* < 0.001), as well as epidermal thickness and the number of epidermal cell layers (*p* < 0.001), in the control group after UVB exposure. Topical application of Cv resulted in a decreased number of sunburnt cells (*p* < 0.001), less inflammation, and less DNA damage (*p* < 0.001). The plant for the study was obtained in Romania. Cv has beneficial effects on the skin when it is applied topically before exposure to UV radiation.

### 2.6. Honeybush (Cyclopia intermedia)

The extract of the honeybush plant is rich in polyphenols; two of the most abundant polyphenols in honeybush are hesperidin and mangiferin [35]. Honeybush extracts have been shown to have chemopreventive properties such as reducing oxidative stress [36], mutagenesis [37], and the development of skin tumors [36]. Petrova et al. [35] conducted a study with 70 female mice randomly divided into seven groups in order to investigate the effect of topical honeybush extract, hesperidin, and mangiferin on the skin of mice before daily exposures to UVB for 10 days. The groups were divided as follows: (1) positive control; (2) negative control; (3) vehicle; (4) “green” honeybush; (5) fermented honeybush; (6) hesperidin; and (7) mangiferin. Outcomes measured included the total polyphenol content and the skin′s response to sunburn. The results showed that fermented honeybush extract had significantly less polyphenol content compared to “green” honeybush extract (*p* < 0.05) and also less flavonoids (*p* < 0.05). The “green” honeybush extract had significantly higher content of hesperidin and mangiferin than the fermented extract (*p* < 0.05). Following daily UVB exposure there was a significant increase in skin cell proliferation and, as well as sunburns indicated by erythema, peeling, thickening, and edema of the skin. The topical “green” honeybush and fermented honeybush extracts led to decreased cell proliferation more so than hesperidin and mangiferin extracts. Additionally, pure hesperidin and mangiferin did not reduce sunburns and thus it can be concluded that honeybush extract is more beneficial than hesperidin and mangiferin alone in protecting the skin from sun damage caused by daily UVB radiation.

### 2.7. Lepidium meyenii (maca)

#### Animal Studies

*Lepidium meyenii* (maca) is a plant of the Peruvian highlands and maca hypocotyl is the edible part of the plant that has been used for its many medicinal properties including increase in fertility [38]. Gonzales-Castaneda et al. [39] investigated whether two different extracts of maca could provide skin protection against UVB radiation. One maca extract was obtained with boiling and the other without boiling. The topical extract was applied to the skin of five mice over three weeks. The results showed that topical treatment with maca extract prevents skin damage caused by UVA, UVB, and UVC exposure. The limitations of this study include small sample size and short duration. However, maca extracts have potential as alternatives in skin protection from UV exposure including sunburns.

## 3. Discussion

### 3.1. Natural Ingredients

Polyphenols are naturally found in fruits and vegetables and in the form of flavonoids, especially in wine, tea, chocolate, coffee, and dietary supplements [40]. Over the last two decades, interest has grown in the use of naturally based ingredients in medicine, which has led to an increase in studies on the effects of polyphenols, as antioxidants, on skin protection from UVA/UVB radiation [41] and cancer prevention. The cost-effective, non-invasive attribute of natural-based ingredients has been an increasing preference of patients as compared to medical procedures or prescriptions when it comes to skin protection in particular [42]. It is important for health care professionals to be informed about the use of natural ingredients, such as polyphenols, in preventing skin damage, sunburns, and other mechanisms that could ultimately lead to the development of skin cancers. Furthermore, knowledge of the evidence is critical in appropriately educating the public about the potential benefits and pitfalls of using natural ingredients.

### 3.2. Polyphenol Bioavailability

Generally, the bioavailability of polyphenols varies depending on the polyphenol forms within the dietary source [43]. Previous studies have attempted to investigate polyphenol absorption after a single dose of polyphenol, either in food/beverage or pill form, by measuring plasma concentrations and/or urinary excretion [43]. Across the multitude of classes of polyphenols, studies have shown a broad range or bioavailability of the different polyphenols [43]. Anthocyanins, for example, are a type of polyphenols commonly found in grapes. Studies have shown that an intake of 150 mg to 2 g of anthocyanins led to low levels of anthocyanins in plasma, indicating poor bioavailability [43]. Bioavailability studies have also been performed on catechins—a type of polyphenol typically found in tea, grapes, and red wine. Studies have shown that bioavailability varies among catechins where EGCG is readily present in plasma after intake, indicating high bioavailability, whereas galloylated catechins were never recovered in urine samples [43]. Across the board, studies have found extensive variance in the bioavailability of polyphenols, which may in part be driven by the nature of the study subjects’ diets and potentially their levels of metabolizing enzymes [43]. Therefore, future studies will have to take additional factors into account when measuring the bioavailability of different polyphenols.

### 3.3. Side Effects

If administered at high doses, some antioxidants may have deleterious effects [2,40]. Research on the toxicity of flavonoids, a type of polyphenol typically found in dietary supplements, suggests that at low concentrations flavonoids have beneficial effects on human cells, while at high concentrations they can have toxic effects and may lead to endothelial injury [40]. Flavonoids exert their toxicity via incorporation into normal human cells, where they lead to increased production of reactive oxygen species (ROS) and thus result in DNA damage and cytotoxicity [40]. Consumption of isoflavones, a type of polyphenol commonly found in soy products, has typically been tied to a number of benefits including a reduced risk of cancer and cardiovascular disease [44,45,46,47]. However, recent studies performed on mice indicate that consumption of the isoflavone genistein may have carcinogenic effects on female reproductive organs due to its high estrogenic potency [48]. The studies reviewed in this article did not reveal any adverse effects from the use of polyphenols they each examined. The dosage application of polyphenols should strive to find a balance between maximizing the protective effects while minimizing the risk of toxic side effects [40,48,49].

### 3.4. Sunscreen and Polyphenolic Cream Comparison

Regular sunscreen protects the skin from the damaging effects of UVR by acting as a chemical or physical barrier that absorbs or reflects UVR, and reduces the amount of UVR that reaches the skin [18]. In contrast, polyphenolic creams do not absorb a significant amount of UVR, suggesting that polyphenolic creams may work through a different mechanism [18]. Additionally, some studies on systemic polyphenols show protective effects on the skin, further supporting the idea that polyphenols may be protective via a separate mechanism [18]. Nevertheless, polyphenolic creams may be a suitable option for individuals with a preference for natural-based ingredients or those who have adverse reactions to regular sunscreen. Since various mechanisms may be involved, it should be explored whether regular sunscreen and polyphenolic cream may have an additive effect if used together. However, one of the limitations of using creams is that individuals tend not to apply them regularly. It has also been reported that individuals do not apply enough sunscreen for adequate protection from damaging UVR and/or they may not apply the topical correctly [50]. Therefore, it is likely that individuals who do not use sunscreen regularly would not use polyphenolic creams either, despite the potential benefits.

### 3.5. Limitations

Interpretation of this review should be considered in light of the limited number of studies that were available. Many of the studies had small sample sizes and a number of studies had poor research design. More studies of high quality are needed to establish the efficacy of different types of polyphenols for prevention of sunburns.

## 4. Materials and Methods

### 4.1. Search Strategy

On 12 July 2016 we searched PubMed for published articles that investigated the effects of polyphenols on sunburn. No limits were placed on the search timeframe. The search combined the keywords “polyphenol” and “sunburn”. No filters were selected. On 22 August 2016 the search was expanded to include the keywords “green tea”, “sunburn”, and “sun protection”.

### 4.2. Selection of Studies

Records were screened by title and/or abstract to exclude studies that did not contribute to answering the question in this review. Inclusion criteria: (1) published in English; and (2) intervention included a polyphenol or a plant-derived polyphenolic extract. Exclusion criteria: (1) in vitro studies; and (2) review articles.

### 4.3. Data Extraction 

Data was extracted from selected studies (Table A1) as follows: (1) polyphenol source; (2) test subjects, country; (3) polyphenol administration; (4) polyphenol content; (5) dosage; (6) study design, duration; (7) control or placebo; (8) major outcome measures; (9) major results; and (10) reference.

## 5. Conclusions

There is increasing evidence that different forms of polyphenols used orally and topically may be beneficial for skin health and, more specifically, for prevention of sunburns. Many naturally occurring products contain polyphenols, including green tea, chocolate, red wine, Romanian propolis, *Calluna vulgaris* extract, grape seeds, honeybush extract, and *Lepidium meyenii* (maca), as reviewed here. Physicians and other health care professionals should be aware of the studies examining the beneficial effects of polyphenols as they could potentially be used as alternatives in skin care and protection from the damaging UV rays. Our research produced a limited number of results for how polyphenols may be used in preventing sunburn. Large-scale clinical studies are needed to assess the use of polyphenols in topical and oral prevention of sunburns. Regardless of the findings reviewed here, prudent sun exposure, the use of sun-protective clothing, and the diligent use of sunscreens are important first-line methods for sun protection.

## Appendix A

**Table A1:** Polyphenol studies summary.

Polyphenol Source	Test Subjects, Country	Polyphenol Administration	Polyphenol Content	Dosage	Study Design, Duration	Control or Placebo	Major Outcome Measures	Major Results	Reference
*HUMAN STUDIES*									
Chocolate	Humans (women), *n* = 74, 20–65 years old, Quebec City, Canada	Systemic (ingested chocolate)	HFC: Epicatechins (mg/day) 10.50 ± 7.54;	30 g of chocolate consumed daily over 12 weeks	2-group, parallel, double-blind, randomized controlled trial	LFC group	Minimal erythema dose (MED) (represents skin sensitivity to UVR)	No significant protective effect of HFC vs. LFC consumption on skin sensitivity to UVR	Mogollon et al., 2014 [25]
			Catechins (mg/day) 11.94 ± 7.91		Treatment groups: G1: High flavonol chocolate (HFC), 30 g/day for 12 + 3 weeks washout period (*n* = 33)				
			LFC: Epicatechins (mg/day) 10.65 ± 7.00; Catechins (mg/day) 14.35 ± 9.28						
					G2: Low flavonol chocolate (LFC), 30 g/day for 12 + 3 weeks wash-out period (*n* = 41)				
Red wine	Healthy male physicians *n* = 15, 28–51 years old, Germany	Topical and systemic	Wine A: 1606 mg/L	Topical: 5 mL of each wine or alcohol at each site for 20 min	Interventional 4-arm study	Ethanol 12% (topical part of the study only)	MED baseline (MED prior to local and systemic wine exposure)	Topical wine application: no significant difference between MED baseline and MED local	Moehrle et al., 2009 [26]
					Spring 2002				
			Wine B: 2052 mg/L		Treatment groups: G1: dressing soaked with 5 mL of each wine or EtOH held in place for 20 min (*n* = 8) G2: oral ingestion of wine A over 40 min (*n* = 5) G3: oral ingestion of wine B over 40 min (*n* = 6) G4: oral ingestion of wine C over 40 min (*n* = 8)				
							MED local (MED before and after application of topical ethanol or wine) MED systemic (MED after oral intake of each wine) (MED = lowest dose at which erythema was produced after 24 h)	Oral consumption of wine: Wine A: no significant difference between MED baseline and MED systemic (*p* = 0.75) Wine B: trend toward increase in MED systemic (*p* = 0.063) Wine C: significantly higher MED systemic (*p* =0.031)	
				Systemic: 6 mL of wine/kg body weight over 40 min at least 7 days between each testing					
			Wine C: 2100 mg/L						
Green tea	Humans, *n* = 21	Topical	Green tea catechins (30%–40%) + other polyphenols (total polyphenol content 40%–50%)	Topical applied to skin	Split body controlled study	Positive control: Placebo lotion (no green tea extract) on the other buttock + UVB	Amount of sunburn cells found on skin biopsies one day after UVB exposure (days 6 and 34) in skin pre-treated with OM24 vs. placebo	Day 6: number of sunburn (apoptotic) cells was unchanged with OM24-pretreatment vs. placebo treated groups	Mnich et al., 2009 [17]
					34 days duration				
					Treatment groups: G1: OM24 applied on one side of buttocks (UV protected skin) 3 times daily for 34 days; UVB exposure on day 5 and 33 about 2 to 3 h after topical applied; Skin biopsies obtained on day 6 and 34			Day 34: number of sunburn cells reduced by 38.9% with OM24-pretreatment (*p* = 0.02) vs. placebo treated groups	
	Data of *n* = 18 analyzed, 21–71 years old, Switzerland	(OM24 topical: contained green tea extract)							
				3 times daily for 34 days					
						Negative control: placebo lotion, no UVB			
			OM24: 4% green tea extract						
Green tea	Humans, 18–50 years old	Topical	1% to 10% green tea polyphenol (GTP);	0.2 mL GTP; concentration 1%–10%	Interventional multi-arm study with each subject as their own control.	Control topical	UV-induced erythema: examined clinically via erythema index at 24, 48 and 72 h post UVR	2.5% GTP: significant reduction in amount of sunburn cells at 24, 48 and 72 h after UVR (*p* < 0.01)	Elmets et al., 2001 [18]
							Number of sunburn cells found in skin biopsies after UVR	Green tea application pre-UV exposure: inhibited erythema formation (*p* < 0.01) and reduced the amount of sunburn cells by 66% (*p* < 0.01)	
					Treatment groups: G1: 0.2 mL of GTP in concentrations ranging from 1% to 10% applied to subjects’ backs (*n* = 6), then exposed to UVR 30 min later				
							DNA damage in skin samples post UVR		
								5% GTP: highest chemoprotective properties compared to EGCG, ECG, EGC, EC	
	*n* = 6, United States (Ohio)		polyphenol constituents = 95% pure, from Japan						
					G2: skin sites on subjects’ back treated with equal concentrations of EGCG, ECG, EC, EGC and 5% GTP (*n* = 6)				
							Determine which purified polyphenol constituent was responsible for chemoprotective properties		
								Topical GTP application led to reduction in	
								Sunburn cells (68% reduction, *p* < 0.01)	
					Biopsies obtained				
								DNA damage (55% reduction, *p* < 0.01)	
Green tea	Humans, 18–65 years old, *n* = 50, United Kingdom	Systemic	Green tea catechins (GTC) 1080 mg/day	3 GTC capsules (180 mg GTC each) + 2 vitamin C capsules (50 mg each) twice daily (stabilizes GTC in gut lumen)	Double-blind randomized placebo-controlled trial 12 weeks duration November 2010 to August 2011	Placebo capsules (maltodextrin)	Difference in MED (defined as sunburn threshold; lowest dose producing visually detectable erythema) at 12 weeks compared to baseline	G1 vs. G2: No significant difference in MED between the GTC group and placebo group (*p* = 0.47)	Farrar et al., 2015 [19]
					Treatment groups: G1: 1080 mg/day GTC + 100 mg/day vitamin C for 12 weeks				
								Within group analysis: G1: No significant difference in MED pre- and post-supplementation (*p* = 0.17) G2: No significant difference in MED at baseline vs. 12 weeks (*p* = 0.12)	
					G2: placebo (maltodextrin capsules identical to GTC and Vitamin C capsules)				
					Upper buttock skin irradiated with UVR (5% UVB, 95% UVA) at baseline and 12 weeks post-supplementation with GTC; 24 h post-UVR, skin visually examined for erythema				
Green tea (GT) and White tea (WT)	Humans, Age not reported, *n* = 10, United States (Ohio)	Topical	GT and WT	2.5 mg/cm^2^	Randomized, double-blind controlled trial	G1: No UV, no treatment G2: UV only, no treatment G3: UV + vehicle	Mean % of CD1a cells (Langerhans cells (LC)) per epidermis unit area in each treatment group – detected via anti-CD1a immunostaining	LC results: G2 and G3 each had 57% reduction in CD1a staining compared to G1 (vehicle = no protection against LC depletion)	Camouse et al., 2009 [20]
					Treatment groups: G1: No UV, no treatment				
					G2: UV only, no treatment			G4 (*p* = 0.002) and G5 (*p* = 0.003) significantly higher % CD1a staining compared to G3	
					G3: vehicle + UV				
					G4: WT + UV				
					G5: GT + UV				
							DNA damage – detected via anti-8-hydroxy-2′-deoxyguanosine (OHdG) staining		
					Topical applied (2.5 mg/cm^2^) to buttock, dried for 15 min, UVR applied at 2 × MED, topical applied again; 72 h later biopsies from 5 sites obtained				
								G4 22% reduction and G5 35% reduction in CD1a staining compared to G1	
								No difference between G4 and G5 in protection against LC depletion (*p* = 0.09)	
								DNA damage results: G2 40% increase, G3 69% increase in 8-OHdG compared to G1	
								G4 and G5: 8-OHdG level not significantly different from G1 (*p* = 0.95 and *p* = 0.12)	
								G4 (*p* = 0.002), G5 (*p* = 0.001) had lower 8-OHdG than G3	
								G4 and G5: no difference in level of protection against UV 8-OHdG formation	
Green tea	Humans, *n* = 20, Chinese women, Age not reported, China	Topical	2%, 3%, 4% and 5% GTE	Applied to 5 sites on dorsal skin 30 min before and 6, 24, 48 h after UVR	Interventional 7-arm study with each subject serving as their own control.	Site 1: no UVR (negative control)	Erythema at various sites assessed via photographs	Erythema: Day 1: Site 2, 3 and 7 developed erythema and with subsequent UVR erythema worsened	Li et al., 2009 [21]
					MED determined 2 weeks before trial:dorsal skin sites exposed to UVR for 4 days:		Thickness of stratum corneum (TSC) and epidermis (TE) (measured by microscopy)		
								Site 5: least amount of erythema	
					Site 1: no UVR				
					Site 2: UVR only (1.5 MED)				
					Site 3: vehicle cream + UVR		Level of cytokeratins (CK): CK 5/6 and CK 16 and metalloproteinases (MMP): MMP-2 and MMP-9 (assessed semi-quantitatively:		
					Site 4: 2% GTE + UVR				
					Site 5: 3% GTE + UVR				
					Site 6: 4% GTE + UVR				
								Day 7: Site 2, 3, and 7—post-inflammatory hyperpigmentation (PIH) Site 4 and 6—moderate PIHSite 5—mild PIH	
					Site 7: 5% GTE + UVR				
							Score 0 = negative		
							Score 1 = slightly positive		
					Site 3 to 7: topicals applied 30 min before UVR and 6, 24, 48 h after last UVR; 7 biopsies obtained 72 h after UVR		Score 2 = moderately positive		
								TSC and TE: Site 2: both TSC and TE increased significantly after UVR (37% and 43%)	
								Site 3: TSC increased by 36% and TE by 42%	
								Compared to site 2, only site 4 and 5 topicals prevented TE increase (*p* < 0.05)	
							Score 3 = strongly positive		
								Compared to site 1, site 2, 3, 6, 7 had increased TSC (*p* < 0.05) while site 4 and 5 were significantly protected	
								CK 5/6 and CK16: Site 2 and 3: CK5/6 and CK16 overexpressed after UVR	
								Site 4, 5, 6, 7: significant protection from UVR, especially site 5	
							Density of CD1a Langerhans cells (LC) in epidermis and dermis (determined via immunohistochemistry and reported as LCs/mm^2^)		
								MMP-2 and MMP-9:	
								Site 1: slight to moderate expression of both	
								Site 2, 3, 7: highest overexpression of moth Site 4, 5, 6: significant decrease in both MMP-2 and MMP-9 (*p* values not reported)	
								LC depletion:	
								Compared to site 1, mean density of LC decreased in site 2 (75%), 3 (64%), 6 (65%), 7 (71%) Site 4 (58% depletion)	
								Site 5 (46% depletion)	
								The difference between site 1 and all others was significant (*p* < 0.05), but not site 5	
**ANIMAL STUDIES**									
Romanian Propolis (RP)	Female Swiss mice, 8 weeks old, *n* = 30, Romania	Topical	RP1 = 3 mg polyphenols/cm^2^	Topical treatment 3 times over 24 h	Interventional 3-arm study	2 control groups: 1. exposed to UVB, no treatment 2. no UVB, no treatment	Amount of sunburn cell formation in epidermis 24 h after UVB exposure as seen on biopsy samples	Pre-treatment with RP extracts: minimized amount of sunburn cells (1.68-fold less for RP1 and 1.77-fold for RP2; *p* < 0.001)	Bolfa et al., 2013 [33]
					3 experiential subsets; 3 groups of treated animals in each				
					Treatment groups:				
					G1: mice treated with vehicle topical				
					G2: mice treated with RP1 topical				
					G3: mice treated with RP2 topical				
								RP post-UVB-treatment: significantly reduced amount of sunburn cell (2.04-fold less for RP1 and 1.98-fold for RP2; *p* < 0.001)	
					Subset 1 (*n* = 10): topical applied before UVB				
					Subset 2 (*n* = 10): topical applied after UVB				
							(sunburn cells identified based on cell membrane shrinkage and nuclear condensation)		
			RP2 = 1.5 mg polyphenols/cm^2^		Subset 3 (*n* = 10): topical applied without exposure to UVB				
								Number of sunburn cells was significantly reduced with RP as compared to the UVB + vehicle group (*p* < 0.05)	
					Skin biopsies obtained 24 h after UVB exposure				
*Calluna vulgaris* (Cv) extract	SKH-1 Hairless Mice, *n* = 50, Romania	Topical	4 mg polyphenols/cm^2^	Apply to skin 30 min before each UVB exposure for 10 days	Interventional 5-arm study	No treatment	Amount of sunburn cell formation after UVB exposure (quantified via histopathological examination of skin biopsies showing apoptosis: pyknotic nuclei and condensed cytoplasm)	UVB (G3) increased number of sunburn cells (3.2 ± 0.76) compared with control (G1) (0.07 ± 0.15; *p* < 0.001)	Olteanu et al., 2012 [34]
					Romania				
					Treatment groups			Pre-treatment with Cv extract significantly reduced the number of sunburn cells compared to groups treated with UVB (G4, 1.80 ± 0.45 vs. G3, 3.2 ± 0.76; *p* < 0.05) Vehicle decreased the number of sunburn cells compared to G4 (*p* < 0.05) Skin lesions were most severe in G3, followed by G5 and G4	
					G1: control (no treatment)				
					G2: vehicle (hydrogel containing Cv extract)				
					G3: UVB only				
					G4: Cv + UVB				
					G5: Cv + vehicle + UVB				
					Extract was applied topically on skin 30 min before each UVB exposure for 10 days; skin fragments excised 24 h after the end of experiment				
Grape seeds -*Vitis vinifera,* Burgund Mare (BM) variety	SKH-1 mice, *n* = 80, 8 weeks old	Topical	BM 2.5 mg polyphenols (PF)/cm^2^	Different doses applied before UVB or 30 min after UVB	Interventional 8-arm study	No treatment G1: Control, G2: vehicle, G3: UV-B only G4: vehicle + UV-B	Number of sunburn cells in skin samples after UVB (identification of apoptotic cells: small, dense nuclei due to nuclear condensation and eosinophilic cytoplasm)	G1, G2: only a few cells underwent normal cell death, with random distribution (0.07% ± 0.15% and 0.74% ± 0.51%)	Filip et al., 2011 [29]
					Treatment groups:				
					G1: Control				
								G3, G4: UVR increased number of sunburn cells significantly (2.26% ± 1.15%; *p* < 0.01)	
					G2: vehicle				
					G3: UV-B only				
								G5: BM 2.5 mg PF/cm^2^ pretreatment reduced number of sunburn cells (1.34% ± 1.17%; 41% inhibition)	
					G4: vehicle + UV-B				
					G5: BM 2.5 mg polyphenols (PF)/cm^2^ + UV-B				
					G6: BM 4 mg PF/cm^2^ + UV-B				
								G6: pretreatment effect on sunburn cells not reported	
					G7: UV-B + BM 2.5 mg PF/cm^2^				
					G8: UV-B + BM 4 mg PF/cm^2^			G7, G8: Both doses of BM extract applied after UV-B reduced sunburn cells (1.21 ± 0.29; 47% inhibition with BM 2.5 mg PF/cm^2^ and 1.07 ± 0.32; 53% inhibition with BM 4 mg PF/cm^2^	
			BM 4 mg PF/cm^2^						
					*n* = 10 in each group				
					Extract applied to skin, 30 min later = UVB exposure; total 10 days.				
					G7 and G8 had UVB exposure and then BM application 30 min later.				
					24 h after last experiment, skin excised for histopathological analysis.				
Honeybush extracts, hesperidin and mangiferin	SKH-1 female mice, 4–6 weeks old, *n* = 70, South Africa	Topical	See results	Topical applied to the skin of SKH-1 mice before daily exposures to UVB light for 10 days	Interventional 4-arm study	Positive, negative and vehicle control groups	Total polyphenol content (determined using Folin Ciocalteu’s phenol reagent)	Fermented honeybush extract: significantly (*p* < 0.05) less total polyphenols (69.916 mg/g) than the ‘‘green’’ honeybush extract (179.618 mg/g) and significantly (*p* < 0.05) less flavonoids	Petrova et al., 2011 [35]
					Treatment groups: G1: ‘‘green’’ honeybush				
					G2: fermented honeybush		Concentration of hesperidin and mangiferin in honeybush extract (determined by high-performance liquid chromatography) Sunburn response of the skin (skin erythema estimated visually daily × 10 days recorded on a scale of degree of damage)		
					G3: hesperidin treatment				
					G4: mangiferin treatment				
					*n* = 10 in each group			Hesperidin and mangiferin: concentrations significantly (*p* < 0.05) higher in ‘‘green’’ honeybush extract (40.742 mg/g and 62.721 mg/g) than in fermented honeybush extract (24.260 and 2.559 mg/g)	
					Each topical was applied to skin, 30 min later skin was exposed to UVB; 10 consecutive days; skin was excised 24 h after last treatment				
								‘‘Green’’ and fermented Honeybush extracts markedly reduced sunburn effects of UVB, while pure compounds hesperidin and mangiferin did not	
EGCG	Mice (wild type) and IL-12 knockout mice, *n* = 60, 6–7 weeks old	Topical	EGCG	1 mg/cm^2^ skin	Interventional case-control study.	No UVB and no EGCG (*n* = 20)	Tumor growth after UVB exposure (skin examined visually for growth of tumors and papillomas); growth >1 mm and if persisted for 2 weeks was recorded	Sunburn cells were maximum 10 h after UVB exposure and decreased thereafter	Meeran et al., 2006 [22]
					Duration: 35 weeks				
					Treatment groups: G1: UVB exposure (*n* = 20) G2: EGCG treatment before UVB exposure (*n* = 20)			EGCG pre-treatment: -significant reduction in number of sunburn cells after UVB-inhibits UVB-induced skin tumorigenesis in WT mice (*p* < 0.001)	
							Detection of apoptotic sunburn cells on skin biopsy samples after H&E stain using light microscopy (sunburn cell: cell membrane shrinkage and nuclear condensation)		
					Topical applied 25–30 min before UVB exposure for total of 10 days; 1 week after last UVB mice got UVB exposure 3 times per week for total 35 weeks; tumor growth evaluated and skin samples obtained				
								-inhibits growth of UVB-induced tumors in WT mice (*p* < 0.001) more than in IL-12 KO mice (*p* < 0.05)	
Green tea	Mice (female CD-1, SKH-1 mice and A/J mice),	Topical and Systemic	Topical: EGCG 49.5% Oral: EGCG 15.1%	Topical: 3.6 mg green tea applied twice weekly	Interventional case-control study	Water	% mice with skin tumors Sunburn lesion formation after UVB exposure for 7 days	Topical GTP: decreased number of tumors by 94%	Conney et al., 1992 [23]
					25 weeks duration			Oral GTP (1.25% and 2.50%) inhibited sunburn formation (*p* < 0.05)	
					Treatment groups:				
					Topical experiment:				
				Oral: 1.25% GT extract and 2.5% GT extract (both were 100% green tea concentration)					
	5–8 weeks old, *n* = 60								
							Skin tumors >1 mm diameter	Oral GTP: decreased number of skin tumors per mouse at 15 weeks (0.3 ± 0.1, *p* < 0.05) and 25 weeks (0.9 ± 0.2, *p* < 0.05)	
					G1: DMBA + TPA (*n* = 30)				
					G2: DMBA + TPA + GTP (*n* = 30)		Subjective measurement of skin lesions based on redness measured daily		
							0 = no lesion		
							1 = barely detectable red lesion		
							2 = moderate red lesion		
							3 = bright red lesion		
					Mice were initiated with 200 mmol DMBA and promoted with 5 mmol TPA twice weekly for 20 weeks; G2 got GTP twice weekly; skin tumor formation assessed after treatment with TPA				
					Oral experiment 1				
					G1: 1.25% green tea + UVB (*n* = 10)				
					G2: 2.50% green tea + UVB (*n* = 10)				
					Two weeks of green tea, then UVB exposure for 7 days while continuing green tea				
					Oral experiment 2: G1: water + UVB + TPA (*n* = 30) G2: 1.25% green tea + UVB + TPA (*n* = 30)				
					Mice consumed green tea extract 1.25% for 2 weeks then exposed to UVB for 10 days. Mice continued GT intake during UVB period and 1 week after. Then, mice only given water and treated topically with TPA.				
*Lepidium meyenii* (maca)	Rats, *n* = 23, Peru	Topical	Boiled and freeze-dried extract total polyphenols: 1.36 ± 0.001 g/100 g	0.05 mg of extract	Interventional 5-arm study with four different treatment groups.	no radiation, no treatment (non-irradiated control)	Epidermal thickness assessed by measuring epidermal height on skin biopsy samples	Aqueous extract of maca resulted in significantly lower epithelial height than pulverized maca (*p* < 0.01) and significantly lowered lower epithelial height than sunscreen application (*p* < 0.01)	Gonzales-Castaneda et al., 2008 [39]
					3 weeks				
					Animal backs were shaved in 5 areas 24 h before UVR exposure; they spent 15 min in front of respective UVR once/week for 3 weeks. (see treatments below). 2 h after last UVR, skin biopsies obtained				
					Treatment groups:				
					G1 (*n* = 5) had these treatments (T) on their backs:				
					T1: aqueous heated extract of maca				
			Non-boiled aqueous extract polyphenol 0.78 ± 0.001 g/100 g						
							Aqueous boiled extract vs. non-boiled pulverized extract in preventing skin damage (quantified by epithelial thickness)		
					T2: non-boiled pulverized extract of maca				
					T3: sunscreen SPF 30				
					T4: distilled water (vehicle)				
					T5: UVC only (irradiated control)				
								Aqueous extract of maca prevents UV-induced skin damage (UVA, UVB, UVC all *p* < 0.01)	
					G2 (*n* = 6): T1: dose 1 maca (0.13 mg/mL) T2: dose 2 maca (0.65 mg/mL) T3: dose 3 maca (1.3 mg/mL) T4: sunscreen 30 SPF T5: irradiated control				
					G3 (*n* = 6): Same treatments as G2 but exposed to UVB once/week for 3 weeks				
					G4 (*n* = 6): same treatments as G2 but exposed to UVC once/week for 3 weeks				
Green tea	Rats, *n* = 24, 12 weeks old, Turkey	Topical	2% EGCG	2% EGCG topical applied 30 min before or after UVA exposure	Interventional 3-arm study	No topical, no UV radiation	Amount of sunburn cells caused by UVA radiation pre and post EGCG topical application (sunburn cells identified by pyknotic nucleus and eosinophilic cytoplasm in biopsy sample)	Group pretreated with EGCG prior to UVA exposure had a statistically significant decrease in sunburnt cells at 24 hours compared with UVA exposure only group (0.67 0.52) *p* < 0.05.	Sevin et al., 2007 [24]
								Group treated with EGCG after UVA exposure had no difference in the development of sunburnt cells compared to the UVA exposure only group.	
					Rats’ backs were shaved and 24 h later rats were placed 30 cm from UVA light. G2 and G3 received 200 mg topical on their back (see below). Biopsies obtained 24 and 72 h after UVA exposure. Histological examination of sunburn cells performed.				
					Treatment groups:				
					G1: UVA exposure				
					G2: UVA exposure + post-treatment EGCG				
					G3: pre-treatment with EGCG + UVA exposure				

Abbreviations: A/J: A/J mouse strain; BM: Burgund mare; CD-1: CD-1 mouse strain; CK: cytokeratin; Cv: *Calluna vulgaris*; DMBA: 7,12-dimethylbenz[α]anthracene; EC: epicatechin; ECG: epicatechin gallate; EGC: epigallocatechin; EGCG: epigallocatechin gallate; G1: group 1; G2: group 2; G3: group 3; G4: groups 4; G5: group 5; G6: group 6; G7: group 7; G8: group 8; GT: green tea; GTP: green tea polyphenol; GTC: green tea catechin; HFC: high flavonol chocolate; IL-12: interlukin-12; LC: Langerhans cell; LFC: low flavonol chocolate; MED: minimal erythema dose; MMP: metalloproteinase; OHdG: anti-8-hydroxy-2′-deoxyguanosine; OM24: green tea extract treatment lotion; PIH: post-inflammatory hyperpigmentation; RP: Romanian propolis; RP1: Romanian propolis 1; RP2: Romanian propolis 2; SKH-1: SKH-1 mouse strain; T1: treatment 1; T2: treatment 2; T3: treatment 3; T4: treatment 4; T5: treatment 5; TE: thickness of epidermis; TPA: 12-*O*-tetradecanoylphorbol-13-acetate; TSC: thickness of stratum corneum; UV: ultraviolet; UVA: ultraviolet A; UVB: ultraviolet B; UVC: ultraviolet C; UVR: ultraviolet radiation; WT: white tea.
